# Rural–Urban Residence and Stroke Risk and Severity in Postmenopausal Women: The Women's Health Initiative

**DOI:** 10.1089/whr.2020.0034

**Published:** 2020-09-09

**Authors:** Shawnita Sealy-Jefferson, Molly Roseland, Michele L. Cote, Amy Lehman, Eric A. Whitsel, Jason Booza, Michael S. Simon

**Affiliations:** ^1^ Division of Epidemiology, College of Public Health, The Ohio State University, Columbus, Ohio, USA.; ^2^ Beaumont Hospital, Oakwood Campus, Dearborn, Michigan, USA.; ^3^ Department of Oncology, Karmanos Cancer Institute Population Studies and Disparities Research Program, Wayne State University School of Medicine, Detroit, Michigan, USA.; ^4^ Center for Biostatistics, Ohio State University, Columbus, Ohio, USA.; ^5^ Department of Epidemiology, University of North Carolina at Chapel Hill, Chapel Hill, North Carolina, USA.; ^6^ Department of Family Medicine and Public Health Sciences, Wayne State University, Detroit, Michigan, USA.

**Keywords:** postmenopausal, rural–urban, stroke risk, stroke severity

## Abstract

***Background:*** The impact of rural–urban residence on stroke risk and poor stroke outcomes among postmenopausal women is unknown.

***Methods:*** We used data from the Women's Health Initiative (WHI) (1993–2014; *n* = 155,186) to test the hypothesis that women who live in rural compared with urban areas have higher stroke risk and worse stroke outcomes than urban women. We used rural–urban commuting area codes to categorize geocoded participant addresses into urban, large rural, or small rural areas. Incident strokes during follow-up were adjudicated by neurologists who used standardized criteria for reviewing brain imaging reports and other medical records and determining stroke subtype. Stroke functional recovery was measured with the Glasgow Stroke Outcomes Scale ascertained from the hospital record. We used univariable and multivariable-adjusted Cox proportional hazards models as well as logistic regression models to test whether rural–urban residence predicted stroke risk and odds of poor stroke outcome.

***Results:*** Among the 155,186 women in our cohort, 2.3% (*n* = 3514) had an incident stroke. We observed a modest reduction in risk of incident stroke among women who lived in urban (adjusted hazard ratio [aHR]: 0.86, confidence interval [95% CI]: 0.71–1.05) and large rural areas (aHR: 0.79, 95% CI: 0.60–1.04) compared with women who lived in small rural areas. In contrast, women who lived in urban compared with large rural areas had a similarly modest increased risk of stroke (aHR: 1.09, 95% CI: 0.89–1.32). Women who lived in urban compared with large rural areas were more likely to have poor stroke outcome (odds ratio [OR]: 1.41, 95% CI: 1.06–1.88), but the association was attenuated after adjustment for covariates (adjusted OR [aOR]: 1.27, 0.93–1.74).

***Conclusions:*** Future studies should confirm and examine the potential pathways of the reported associations among postmenopausal women.

## Introduction

Stroke is the foremost cause of long-term adult morbidity and the fifth leading cause of death in the United States. Nearly 800,000 strokes occur each year^1,2^ costing an estimated $34 billion/year in health care, medication, and missed days of work.^3,4^ Stroke primarily affects older populations, with a doubling of risk for each additional decade of life after 55 years of age.^5^ It has been projected that the number of people who experience a stroke will increase by 3.4 million people by the year 2030, which represents a significant burden of stroke among U.S. residents.^4^

The menopausal transition has been identified as a period when many women develop risk factors for cardiovascular disease, including stroke.^6^ Women have a higher burden of stroke, likely because stroke risk increases with age, and women have a longer life expectancy than men. The majority of strokes occur in women aged >70 years, who are more likely to live alone, have constrained finances, are socially secluded, and have more comorbid diseases.^4^ After stroke, women are also half as likely as men to have independence in terms of activities of daily living.^7^

One fifth of the U.S. population lives in isolated rural areas,^8^ which are often fraught with longer travel times to access health care.^9^ Stroke risk is greatest in the rural southeast and coastal areas of the United States,^10^ but there are limited data on geographic differences in stroke risk and outcomes among older women. Stroke mortality is 30% higher in rural compared with urban areas in the United States, and preliminary evidence suggests that this disparity is due to a higher incidence rather than a higher case fatality among stroke patients.^11,12^ If incidence is indeed the driver of the rural mortality penalty, primary prevention may be effective at reducing the burden of stroke mortality in rural areas.^11^

There are also rural–urban disparities in acute stroke care that can be attributed to structural related barriers (organizational features, provider, facility, and access to care) and patient-level factors (eligibility for treatment and disease severity).^13^ There are striking and growing disparities in treatment for acute stroke in rural versus urban areas.^13^ Although rural residents are more likely to have a usual source of health care^14^ (“a place to which they usually go when they are sick”^15^), they are still less likely to have a local hospital and physician,^16^ more likely to be uninsured,^17^ and tend to report fewer annual health care visits.^18^ Such disparities may have an impact on stroke incidence in rural and urban areas given that the availability of health care resources has a strong influence on risk factor prevalence,^19^ awareness, treatment, and control.^20^

Most of the research on geographic disparities in stroke has been focused on understanding the higher stroke burden in the southeastern region (or so-called “Stroke Belt”) of the United States.^21^ Although evidence of increased stroke mortality in this area has existed for decades,^19^ recent results from an ecological study suggest racial and urban–rural disparities in mortality rates among those residing outside of the Stroke belt.^22^ Conversely, evidence of geographic differences in stroke incidence is lacking. Using the National Health and Nutrition Examination Survey, researchers reported higher stroke incidence rates in southeastern compared with northeastern states, but inconsistent patterns were found among other regions.^23^ Results from the Reasons for Geographic and Racial Differences in Stroke study found that stroke risk was 23% higher in large rural towns, and 30% higher in small rural/isolated areas, compared with urban areas.^11^ Importantly, stroke patients from rural areas may have higher comorbidity and risk factor profiles,^24^ receive lower quality stroke care,^25,26^ and delayed consultation with stroke neurologists, all of which could result in poor poststroke outcomes.

To extend the literature on rural–urban residence on stroke risk and outcomes, we examined whether rural–urban residence was associated with stroke risk and poststroke outcomes among a large cohort of racially/ethnically diverse postmenopausal women from the Women's Health Initiative (WHI). We hypothesized that residence in small rural areas, compared with large rural and urban areas would be a significant predictor of stroke risk and poor stroke outcomes.

## Materials and Methods

### Study population

Details of the WHI have been published.^27–29^ In brief, a total of 161,808 postmenopausal women, from 40 U.S clinical centers, across 24 states and the District of Columbia, were randomized to overlapping dietary modification, calcium/vitamin D, and hormone therapy clinical trials (*n* = 68,132) or enrolled in an observational study (*n* = 93,676), between 1993 and 1998. Women were eligible to participate if they were between 50 and 79 years of age, willing to give informed consent, and expected to survive and not relocate during the next 3 years.

### Rural–urban classification

We used Rural–Urban Commuting Area (RUCA) codes that classify all U.S. Census tracts into one of 10 main categories, as well as 33 subcategories based on secondary commuting flows.^30^ U.S. Census tracts provide more granular information about neighborhoods than larger county definitions.^11^ Study participant addresses were geocoded as previously described,^31–33^ and assigned a tract-level, RUCA code based on a reference document from the United States Department of Agriculture.^30^ Our analytic sample included 155,186 women whose addresses were linkable to the RUCA coding scheme ≤2 years after they were randomized in the clinical trials or enrolled in the observational study.

We grouped the RUCA codes into four categories: (1) urban or “metropolitan” (codes: 1.0, 1.1, 2.0, 2.1, 3.0, 4.1, 5.1, 7.1, 8.1, 9.1, and 10.1); (2) large rural city/town or “micropolitan” (codes: 4.0, 4.2, 5.0, 5.2, 6.0, and 6.1) (3) small rural town (codes: 7.0, 7.2, 7.3, 7.4, 8.0, 8.2, 8.3, 8.4, 9.0, and 9.2), and (4) isolated small rural town (codes: 10.0, 10.2, 10.3, 10.4, 10.5, and 10.6). Given the small number of study participants in the isolated small rural town, we combined this category with small rural town category for the analysis. RUCA's small census tract scale is able to uniquely capture fine spatial variation in rurality, allowing RUCA to classify individuals by geographic region more precisely than other county-based classification schemes.^34^ In addition, the RUCA classification emphasizes commuting flows, which are important to consider in the context of stroke diagnosis and treatment, since “communities to which persons flow (for employment) may also be places where they receive health care.^34^”

### Covariates

Potential confounders (variables that were likely to be associated with both outcome and exposure but not on the causal pathway linking exposure to outcomes) were identified from the literature,^11^ and included the following variables ascertained from the baseline interview: age, race/ethnicity (black, white, or other), income (<$20k, $20–34,999, $35–74,999, and $75k+), education (none/some high school, high school/general equivalency degree, or more than high school), smoking status (former, current, or never), physical activity (metabolic equivalents/week), living alone (yes/no), history of medications to treat hypertension (yes/no), and history of atrial fibrillation (ever/never).

### Stroke incidence and severity

Follow-up for incident stroke was performed at 6-month intervals for the clinical trials and every year for the observational study through 2010. Study participants in the clinical trials had at least yearly follow-up clinic visits, and observational study participants provided follow-up information by mail. Women with self-reported stroke history at baseline were excluded from these analyses. The majority of strokes occurring during follow-up were adjudicated by neurologists who used standardized criteria for reviewing brain imaging reports and other medical records and determining stroke subtype. However, for our analytic sample, all strokes were adjudicated. Strokes were classified as ischemic (thrombotic or embolic occlusion of a cerebral artery or lacunar infarction not related to a procedure), hemorrhagic (subarachnoid, intracerebral, or other undetermined intracranial hemorrhage not related to procedure), or unknown stroke type when a stroke was documented, but type could not be determined.^35^

Stroke functional recovery was measured with the Glasgow Stroke Outcomes Scale ascertained from the hospital record, and was categorized as (1) good recovery (able to resume work or school), (2) moderate disability (able to live independently, but not able to resume work or school), (3) severe disability (able to follow commands, unable to live independently), (4) vegetative survival, (5) death, and (6) unable to assess.

### Statistical analysis

Our analytic sample included 155,187 women for whom we were able to assign a RUCA code to their address. One woman had a stroke on day 0, so was not included in our Cox models, leaving 155,186 women in our analytic sample. We calculated frequencies of baseline sociodemographics, clinical factors, and stroke risk factors, overall and by incident stroke status. We also summarized the clinical characteristics of women diagnosed with a stroke, according to rural–urban classification. We used univariable and multivariable-adjusted Cox proportional hazards models to quantify the association between RUCA code and incident stroke.

For all models, participant age used as the time scale, and the baseline hazard was stratified by trial membership. The Glasgow outcome scale was dichotomized as good recovery (1, 2) versus poor recovery (3, 4, 5). We used univariable and multivariable logistic regression models to quantify the association between urban–rural residence and stroke outcome, among women who had available data on stroke recovery. Analyses were performed using SAS/STAT software, Version 9.4 of the SAS System for Windows (SAS Institute, Inc., Cary, NC). Finally, we take heed to recent calls to retire statistical significance, in favor of more detailed and nuanced statistical analyses and interpretation of results, recognizing that *p*-values and decisions regarding which research ideas should be explored further have no association.^36^

## Results

Of 155,186 study participants, 2.3% (*n* = 3514) had an incident stroke (Table 1), during an average follow-up of 8.6 years (maximum follow-up = 10 years). A higher proportion of women with a stroke had worse socioeconomic status than women without incident stroke during follow-up. The distribution of biologic and behavioral risk factors for stroke were as expected in women with and without incident stroke. More women with an incident stroke reported fair/poor self-rated health (16%) than their peers without an incident stroke (9%). According to the RUCA coding scheme, 93% (*n* = 144,937) of WHI participants lived in urban, 4% (*n* = 5923) large rural, and 3% (*n* = 4321) small/isolated rural areas.

**Table 1.** Demographic Characteristics of Study Participants in Women's Health Initiative (*n* = 155,186) and Stratified by Incident Stroke Status, 1993–2010

Characteristic	Total sample, N = 155,186 (%)	No stroke, N = 151,672 (%)	Stroke, N = 3514 (%)
Age			
50–59	50,709 (33%)	50,300 (33%)	409 (12%)
60–69	70,015 (45%)	68,533 (45%)	1482 (42%)
70–79	34,462 (22%)	32,839 (22%)	1623 (46%)
Race			
Black	13,204 (9%)	12,841 (8%)	363 (10%)
White	128,831 (83%)	125,907 (83%)	2924 (83%)
Other	12,749 (8%)	12,538 (8%)	211 (6%)
Education			
None/some HS	8185 (5%)	7923 (5%)	262 (7%)
HS/GED	26,594 (17%)	25,926 (17%)	668 (19%)
>HS	58,286 (38%)	56,915 (38%)	1371 (39%)
Any insurance			
No	6815 (4%)	6712 (4%)	103 (3%)
Yes	146,875 (95%)	143,500 (95%)	3375 (96%)
Income			
<$20,000	24,192 (16%)	23,316 (15%)	876 (25%)
$20k–$34,999	35,261 (23%)	34,305 (23%)	956 (27%)
$35k–$74,999	58,465 (38%)	57,371 (38%)	1094 (31%)
$75k+	26,863 (17%)	26,516 (17%)	347 (10%)
Hypertension			
Never	96,873 (62%)	95,364 (63%)	1509 (43%)
Untreated	11,904 (8%)	11,554 (8%)	350 (10%)
Treated	37,938 (24%)	36,520 (24%)	1418 (40%)
High cholesterol pills			
No	125,355 (81%)	122,689 (81%)	2666 (76%)
Yes	20,731 (13%)	20,111 (13%)	620 (18%)
Treated diabetes			
No	148,245 (96%)	145,117 (96%)	3128 (89%)
Yes	6804 (4%)	6424 (4%)	380 (11%)
Relative had a stroke			
No	90,097 (58%)	88,305 (58%)	1792 (51%)
Yes	56,205 (36%)	54,715 (36%)	1490 (42%)
Don't know	7822 (5%)	7619 (5%)	203 (6%)
No. of relatives who had a stroke			
0	90,097 (58%)	88,305 (58%)	1792 (51%)
1	46,291 (30%)	45,150 (30%)	1141 (32%)
2	8145 (5%)	7861 (5%)	284 (8%)
3	1129 (1%)	1082 (1%)	47 (1%)
4 or more	427 (<1%)	415 (<1%)	12 (<1%)
Atrial fibrillation			
No	145,757 (94%)	142,677 (94%)	3080 (88%)
Yes	6820 (4%)	6472 (4%)	348 (10%)
Hormone therapy			
Never used E alone or E + P	67,955 (44%)	66,274 (44%)	1681 (48%)
Past user or E alone or E + P	24,888 (16%)	24,162 (16%)	726 (21%)
Current user of E	35,110 (23%)	34,347 (23%)	763 (22%)
Current user of E + P	27,104 (17%)	26,763 (18%)	341 (10%)
BMI (categorized)			
<25	54,214 (35%)	53,102 (35%)	1112 (32%)
25–29	53,558 (35%)	52,318 (34%)	1240 (35%)
30+	46,191 (30%)	45,059 (30%)	1132 (32%)
Alcohol			
Nondrinker	45,722 (29%)	44,450 (29%)	1272 (36%)
Former drinker	90,360 (58%)	88,543 (58%)	1817 (52%)
Current drinker	18,003 (12%)	17,607 (12%)	396 (11%)
Smoking			
Never smoker	78,321 (50%)	76,579 (50%)	1742 (50%)
Past smoker	64,270 (41%)	62,871 (41%)	1399 (40%)
Current smoker	10,554 (7%)	10,229 (7%)	325 (9%)
Self-rated health			
Excellent/very good	89,456 (58%)	87,911 (58%)	1545 (44%)
Good	50,771 (33%)	49,397 (33%)	1374 (39%)
Fair/poor	13,947 (9%)	13,382 (9%)	565 (16%)
Physical activity (MET/week)			
None	23,381 (15%)	22,741 (15%)	640 (18%)
>0–3.75	21,637 (14%)	21,087 (14%)	550 (16%)
3.75–8.75	30,421 (20%)	29,728 (20%)	693 (20%)
8.75–17.5	33,480 (22%)	32,771 (22%)	709 (20%)
≥17.5	38,950 (25%)	38,211 (25%)	739 (21%)
RUCA class			
Urban	144,937 (93%)	141,669 (93%)	3268 (93%)
Large rural	5928 (4%)	5799 (4%)	129 (4%)
Small rural	2331 (2%)	2271 (1%)	60 (2%)
Isolated small rural	1990 (1%)	1933 (1%)	57 (2%)
WHI Clinical Trial Membership			
Observational study	90,117 (58)	88,126 (58)	1991 (57)
E-alone trial	6963 (4)	6734 (4)	229 (7)
E+P trial	11,471 (7)	11,177 (7)	294 (8)
DM trial	38,966 (25)	38,176 (25)	790 (22)
E-alone/E+P trial+DM	7669 (5)	7459 (5)	210 (6)

DM, dietary modification; HS, high school; GED, general education diploma; RUCA, rural–urban commuting area; WHI, Women's Health Initiative.

The mean age at stroke diagnosis was slightly lower for women who lived in large rural areas, compared with those who lived in urban and small rural areas (Table 2). There was a higher proportion of hemorrhagic strokes in women who lived in large rural areas (23%) than urban (15%) or small rural areas (15%). A higher proportion of women who lived in small rural areas had good poststroke recovery (29%) compared with women who lived in urban (27%) and large rural areas (27%). However, a higher proportion of women who lived in large rural areas died as a result of their stroke (11%) than women who lived in urban or small rural areas (both 9%).

**Table 2.** Clinical Characteristics of Women with Incident Stroke by Rural–Urban Residence: Women's Health Initiative (*n* = 3514), 1993–2010

	Rural–urban category			
Characteristic	Total n* = 3514, *N (%)	Urban, N* = 3268, *N (%)	Large rural, N* = 129, *N (%)	Small rural, N* = 117, *N (%)
Approximate age at diagnosis				
Mean (SD)	73.3 (6.8)	73.3 (6.8)	72.2 (6.5)	73.6 (6.2)
Min, max	50.7, 89	50.7, 89	56.9, 85.7	58.3, 85.1
Stroke type				
Ischemic	2654 (76)	2472 (76)	94 (73)	88 (75)
Hemorrhagic	548 (16)	501 (15)	30 (23)	17 (15)
Other/missing	312 (9)	295 (9)	5 (4)	12 (10)
Glasgow Stroke Outcome Scale				
1: Good recovery	961 (27)	892 (27)	35 (27)	34 (29)
2: Moderately disabled	896 (26)	821 (25)	45 (35)	30 (26)
3: Severely disabled	856 (24)	808 (25)	20 (16)	28 (24)
4: Vegetative survival	18 (1)	18 (1)	0	0
5: Deceased	323 (9)	298 (9)	14 (11)	11 (9)
6: Unable to assign category/missing	460 (13)	431 (13)	15 (12)	14 (12)
Stroke outcome (*n* = 3054):				
Poor (Glasgow Stroke Outcome Scale = 3, 4 or 5)	1197 (39)	1124 (40)	34 (30)	39 (38)
Good/moderate (Glasgow Stroke Outcome Scale = 1 or 2)	1857 (61)	1713 (60)	80 (70)	64 (62)

We observed a modest reduction in risk of incident stroke among women who lived in urban (adjusted hazard ratio [aHR]: 0.86, confidence interval [95% CI]: 0.71–1.05) and large rural areas (aHR: 0.79, 95% CI: 0.60–1.04) compared women who lived in small rural areas (Table 3). Nevertheless, a risk difference ranging from a 29% decreased risk to 5% increased risk for incident stroke comparing women residing in urban to small rural areas, and between a 40% decreased risk to a 4% increased risk comparing women who lived in large to small rural areas, is also compatible with our data, given our assumptions. In contrast, women who lived in urban compared with large rural areas had a similarly modest increased risk of stroke. However, a risk difference ranging from an 11% decreased to a 32% increased risk is also compatible with our data, given our assumptions (aHR: 1.09, 95% CI: 0.89–1.32).

**Table 3.** Relationship Between Rural–Urban Residence and Incident Stroke: Women's Health Initiative (*n* = 155,186), 1993–2010

Rural–urban comparisons	Unadjusted, HR (95% CI)	Adjusted, HR (95% CI)
Urban	0.84 (0.70–1.01)	0.86 (0.71–1.05)
Large rural	0.81 (0.63–1.03)	0.79 (0.60–1.04)
Small rural (reference)	1.00	1.00
Urban	1.05 (0.88–1.25)	1.09 (0.89–1.32)
Large rural (reference)	1.00	1.00

Adjustments: race, smoking, income, education, physical activity, living alone, hypertension, treatment for diabetes, and atrial fibrillation; age was used as the time scale.

CI, confidence interval; HR, hazard ratio.

Stroke outcome data were available for 3024 women. In univariable models we observed lower probability of poor stroke outcome (Glasgow score of 3, 4 or 5) among women who lived in urban and large rural compared with small rural areas (Table 4). However, a 26% lower odds to a 37% increased probability of poor stroke outcome in urban compared with small rural areas is also compatible with our data, given our assumptions. In contrast, for women who lived in large rural compared with small rural areas, a proportion difference ranging from a 63% reduction to a small 7% increased odds is also compatible with our data, given our assumptions. Similarly, our adjusted results for women who lived in large rural compared with small rural areas suggested a 13% decreased risk; however, a risk difference ranging from a 45% reduction in risk to a 36% increased risk is also compatible with our data. Similarly, our adjusted point estimates for women who lived in urban compared with small rural areas had 10% increased risk of poor stroke outcome; however, a risk difference from a 22% decreased risk to a sizeable 55% increased risk is also compatible with the data, given our assumptions. Finally, in univariable models, those who lived in urban compared with large rural areas, were more likely to have poor stroke outcome (odds ratio [OR]: 1.41, 95% CI: 1.06–1.88), but the association was slightly attenuated after adjustment for covariates (adjusted OR [aOR]: 1.27, 0.93–1.74). However, a difference in odds for women who lived in urban compared with large rural areas ranging from a 7% reduction to a sizeable 74% increase in odds of poor stroke outcome, after adjusting for covariates, is also compatible with our data, given our assumptions.

**Table 4.** Relationship Between Rural–Urban Residence and Stroke Outcome: Women's Health Initiative (*n* = 3054), 1993–2010

Rural–urban comparisons	Unadjusted, OR (95% CI)	Adjusted, OR (95% CI)
Urban	1.02 (0.74–1.37)	1.10 (0.78–1.55)
Large rural	0.71 (0.47–1.07)	0.87 (0.55–1.36)
Small rural (reference)	1.00	1.00
Urban	1.41 (1.06–1.88)	1.27 (0.93–1.74)
Large rural (reference)	1.00	1.00

Adjustments: age, race, smoking, income, education, physical activity, living alone, hypertension, treatment for diabetes, atrial fibrillation, and trial membership.

OR, odds ratio.

## Discussion

The National Institutes of Health includes rural residents in their definition of health disparity populations,^37^ because disease prevalence and mortality rates are higher in these areas than in the overall U.S. population.^38^ Our adjusted point estimates suggests a possible risk difference ranging from a sizeable 29% reduced risk to a relatively small 5% increased risk of stroke in postmenopausal women who lived in urban compared with small rural areas,. For women who lived in large rural compared with small rural areas, our results suggest a possible risk difference ranging from a 40% decreased risk to a small 4% increased risk. We found evidence of a difference in odds ranging from a 10% reduced probability to a 108% increase in odds of poor stroke outcome among women who lived in urban compared with large rural areas.

Our results are similar to those reported in another study examining rural–urban residence and stroke risk, which used data from 18,705 men and women enrolled in the REGARDS study. Specifically, they found that compared with urban residents, those who lived in large rural had 28% increased risk of stroke, but risk differences ranging from a 4% reduction in risk to a sizeable 44% increased risk was also compatible with their data. In comparing stroke risk among those who lived in small rural areas to urban, they reported the former had a 19% higher risk, although the range of 6% decreased risk to 50% increased risk is also compatible with their data. They also reported point estimates suggesting higher case fatality for individuals living in large rural compared with urban areas (OR: 1.13, 95% CI: 0.63–2.01) and point estimates suggesting a protective effect for small rural area residents (OR: 0.70, 95% CI: 0.33–1.44); however, the uncertainty around all of their estimates was non-negligible. We add to this literature evidence from a large longitudinal cohort study of postmenopausal women, which suggests that women who live in small rural compared with large rural and urban areas, and women who live in urban compared with large rural areas may have higher stroke risk and worse stroke severity. Future research is needed to confirm our findings and examine potential mediating pathways.

Intrarural disparities in mortality exist, with rural areas of modest population size and spatially close to urban areas, having greater mortality burden than the most rural areas.^39^ In our study, a higher proportion of women who resided in large rural areas had a hemorrhagic stroke (23%) compared with women who lived in urban and small rural areas (both 15%). The prevalence of hemorrhagic strokes range from 6.5% to 16.6%.^40^ Future studies should explore intrarural differences in stroke type. Potential reasons for our modest results regarding a rural penalty for stroke risk and poor stroke outcome could be insufficient variability in rural–urban residence, and/or that rural residents commonly receive their stroke care in urban settings,^41^ or have telestroke care in rural care settings,^42,43^ which could reduce the difference in quality of care by rural–urban residence.

Our findings may also be the result of successful stroke care quality improvement programs, such as the Paul Coverdell National Acute Stroke Program, the American Heart Association and the American Stroke Association, and the Joint Commission's Primary Stroke Center Certification program, initiatives that sought to organize and coordinate acute stroke care in rural hospitals.^44^ Furthermore, evidence suggests that rural populations are more likely to have a usual source of health care than their urban counterparts.^44^ It is unclear why women who lived in urban compared with large rural areas may have higher stroke risk and worse poststroke outcomes.

Future research with larger study populations should be able to confirm this association and identify intervening pathways. In interpreting our results, the following limitations should be considered. First, the WHI is not a population-based sample, and study participants had higher socioeconomic position and were healthier than the general U.S. population. However, the racial/ethnic composition of the WHI is comparable with the U.S. population. In our analysis subsample, 9% of the participants had missing stroke type and stroke outcome data. The heterogeneity of our sample in terms of rurality of residence was low, as was the risk of stroke in our cohort, both of which may have decreased our power to detect rural–urban differences in stroke risk and outcomes. Given the hypothesis generating nature of this research, we did not adjust for multiple comparisons.

Our study has several strengths that extend the current literature. First, to our knowledge, ours is the largest study of the influence of rural–urban residence on stroke risk and outcome in postmenopausal women.^45^ We were also able to control the analysis for lifestyle factors not available in many stroke databases. Another strength of our work is the geographic heterogeneity of the WHI, and that we had 3514 stroke events, which allowed us to estimate the impact of rural–urban residence on stroke risk among postmenopausal women. WHI is a rich large data set to study stroke in aging women, given the long-term follow-up and large study population.^46^

## Summary

Our results suggest that postmenopausal women who live in small rural compared with large rural and urban areas may have higher stroke risk and worse stroke severity, and that women who live in urban compared with large rural areas may have higher stroke risk and worse stroke outcomes. Since most people will survive their stroke, poststroke disability is the ultimate challenge stroke patients and their families face, as two thirds of stroke patients have a severe/moderate or mild disability.^47,48^ To address the projected human, economic, and societal burden of stroke, it is imperative that future research identify novel risk factors for stroke risk and poor stroke outcome among higher risk groups, including geographic areas most affected.

## Abbreviations Used:

aHR: adjusted hazard ratio
aOR: adjusted odds ratio
CI: confidence interval
OR: odds ratio
RUCA: rural–urban commuting area
WHI: Women's Health Initiative
